# Dietary acid load and chronic kidney disease among adults in the United States

**DOI:** 10.1186/1471-2369-15-137

**Published:** 2014-08-24

**Authors:** Tanushree Banerjee, Deidra C Crews, Donald E Wesson, Anca Tilea, Rajiv Saran, Nilka Rios Burrows, Desmond E Williams, Neil R Powe

**Affiliations:** 1 Department of Medicine, University of California, San Francisco, CA, USA; 2 Division of Nephrology, Department of Medicine, Johns Hopkins University School of Medicine, Baltimore, MD, USA; 3 Welch Center for Prevention, Epidemiology and Clinical Research, Johns Hopkins Medical Institutions, Baltimore, MD, USA; 4 Texas A&M College of Medicine and Scott and White Healthcare, Texas, USA; 5 Kidney Epidemiology & Cost Center, University of Michigan, Ann Arbor, MI, USA; 6 Division of Nephrology, Department of Medicine and Kidney Epidemiology & Cost Center, University of Michigan, Ann Arbor, MI, USA; 7 Centers of Disease and Control and Prevention, Atlanta, GA, USA; 8 Department of Medicine, San Francisco General Hospital, San Francisco, CA, USA

**Keywords:** Acidosis, Albuminuria, Chronic kidney disease, NHANES (National Health and Nutrition Examination Survey), Nutrition

## Abstract

**Background:**

Diet can markedly affect acid-base status and it significantly influences chronic kidney disease (CKD) and its progression. The relationship of dietary acid load (DAL) and CKD has not been assessed on a population level. We examined the association of estimated net acid excretion (NAE_es_) with CKD; and socio-demographic and clinical correlates of NAE_es_.

**Methods:**

Among 12,293 U.S. adult participants aged >20 years in the National Health and Nutrition Examination Survey 1999–2004, we assessed dietary acid by estimating NAE_es_ from nutrient intake and body surface area; kidney damage by albuminuria; and kidney dysfunction by eGFR < 60 ml/min/1.73m^2^ using the MDRD equation. We tested the association of NAE_es_ with participant characteristics using median regression; while for albuminuria, eGFR, and stages of CKD we used logistic regression.

**Results:**

Median regression results (β per quintile) indicated that adults aged 40–60 years (β [95% CI] = 3.1 [0.3–5.8]), poverty (β [95% CI] = 7.1 [4.01–10.22]), black race (β [95% CI] = 13.8 [10.8–16.8]), and male sex (β [95% CI] = 3.0 [0.7- 5.2]) were significantly associated with an increasing level of NAE_es_. Higher levels of NAE_es_ compared with lower levels were associated with greater odds of albuminuria (OR [95% CI] = 1.57 [1.20–2.05]). We observed a trend toward greater NAE_es_ being associated with higher risk of low eGFR, which persisted after adjustment for confounders.

**Conclusion:**

Higher NAE_es_ is associated with albuminuria and low eGFR, and socio-demographic risk factors for CKD are associated with higher levels of NAE_es_. DAL may be an important target for future interventions in populations at high risk for CKD.

## Background

Diet can markedly affect the acid-base status [1-4] and it significantly influences the chronic kidney disease (CKD) and its progression [5-8]. Dietary acid load (DAL) is determined by the balance of acid-inducing foods which is rich in animal proteins (such as meats, eggs, and cheese) and base-inducing foods which is rich in fruits and vegetables (such as raisins, apples, peaches, spinach, and cauliflower). Intake of acid-inducing foods in high amounts for a sufficient period of time can induce metabolic acidosis [9,10]. Acid-inducing diets are believed to impact the kidney via tubular toxicity of elevated ammonium concentrations and activation of the renin-angiotensin system [11,12]. With increased dietary acid load, production of ammonia is increased in the proximal tubule and H^+^ excretion is increased distally to augment overall acid excretion [13,14].

Prior studies have demonstrated that dietary acid-loading increased and base-loading decreased angiotensin II, endothelin-1, and aldosterone –mediated kidney injury [5,6]. Some small translational studies in patients with early CKD demonstrated that oral alkali or base-inducing fruits and vegetables (e.g. raisins, apples, spinach) decreased urinary endothelin-1, aldosterone, and markers of tubulointerstitial injury in addition to slowing GFR decline [5,11]. Although a study by Scialla et al. [15] conducted among African Americans with hypertension-attributed nephropathy found that higher net endogenous acid production was associated with faster GFR decline and Kanda et al. [16] found similar results in a cohort with older CKD adults, the degree to which DAL is associated with risk of CKD in large, representative populations has not been explored. Furthermore, the relation of DAL with characteristics of persons at risk for CKD is largely unknown. Understanding these relations could provide a foundation for dietary interventions in CKD. Therefore, we undertook a population-based study to investigate whether dietary acid content, which we quantified as net acid excretion (NAE_es_) estimated from 24-hr dietary recall, is associated with advanced stages of CKD or albuminuria. We also investigated the relation between socio-demographic and clinical characteristics and DAL.

## Methods

### Study design and population

**Figure 1:**
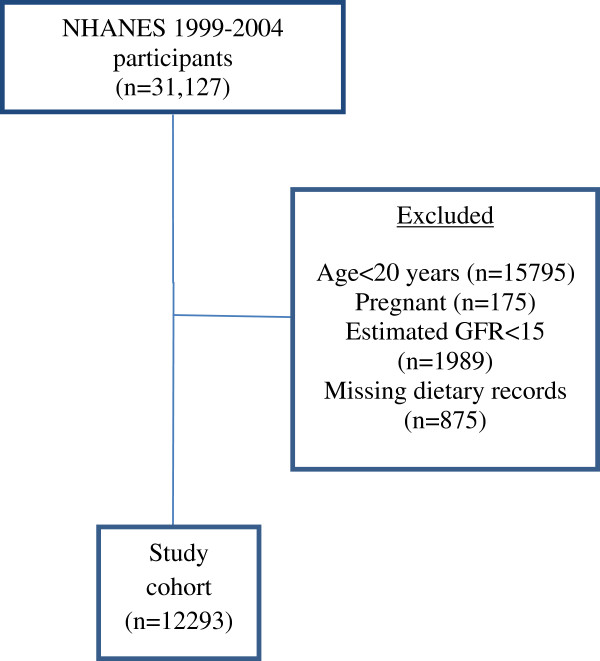
Summary of reasons for participant exclusion from study population.

We performed a cross-sectional analysis of non-institutionalized United States adult participants of the National Health and Nutrition Examination Survey (NHANES) years 1999–2000, 2001–2002, and 2003–2004. Although the total numbers of participants for these study years was 31,126, our study was limited to the 12,293 subjects who were at least 20 years of age, underwent a Mobile Examination Center (MEC) examination, provided a dietary recall interview, had an estimated glomerular filtration rate (eGFR) ≥ 15 ml/min/1.73m^2^ calculated by using the Modification of Diet in Renal Disease (MDRD) Study equation [17], and were not pregnant (Figure 1).

### Socio-demographic and clinical measurements

Medical and demographic data were collected through a standardized survey conducted at participants’ homes, followed by a medical examination and laboratory testing that occurred in the MEC.

Socio-demographic factors were assessed during the interview. Racial/ethnic categories were self-reported by participants and assigned by NHANES as: non-Hispanic white (NHWs), non-Hispanic black (NHBs), Mexican Americans, and others (Asians, Native Americans, other Hispanics and those of unknown race/ethnicity). Self-reported information on socioeconomic status (SES) (education and income), and routine site of health care were obtained during the interview portions of the surveys. Income was assessed using the poverty income ratio (PIR), which is a ratio of household income to household poverty level [18].

Diabetes was defined by self-report of the condition or measured hemoglobin A_1c_ (A_1c_) ≥ 6.5% [19]. Hypertension was defined by self-report of being told by healthcare providers of having the condition, a measured average systolic blood pressure (BP) of ≥140 mm Hg, or average diastolic BP of ≥90 mm Hg or reported use of antihypertensive medications [20]. Subjects were identified as having self-reported cardiovascular disease if they answered “yes” to the question “Have you ever been told by a doctor that you have/had coronary heart disease, myocardial infarction or heart attack, cerebrovascular accident/stroke, angina, or congestive heart failure?” Smoking status was categorized as “current”, “past”, or “never” (no prior) cigarette use.

### Dietary recall interview and NAE_es_

The dietary intake data collected in the NHANES were used to estimate the types and amounts of foods and beverages consumed during the 24-hour period prior to the interview (midnight to midnight), and to estimate intake of energy, nutrients, and other food components from those foods and beverages. The first day of the dietary interview component was collected in the MEC while the second day of the interview component was collected over the telephone 3 to 10 days later, and only in the 2003–2004 survey. We therefore carried out our analysis based on the first day of the dietary interview component. The non-bicarbonate anions (protein, phosphorus) intake and the mineral cations (potassium, magnesium, calcium) intake of foods consumed by participants were derived from the dietary intake data in NHANES. Potential renal acid load (PRAL) of foods consumed by the participants was calculated from estimated nutrient intake data derived from the NHANES dietary recall questionnaire, using the calculation model developed by Remer and Manz [*PRAL (mEq/d) = 0.49*∗*protein(g) + 0.037*∗*phosphorus(mg)]- 0.021*∗*potassium(mg)-0.026*∗*magnesium(mg)-0.0125*∗*calcium(mg)]*[1]. Net acid excretion (NAE_es_) was estimated as *NAE*_*es*_*(mEq/d) = PRAL + organic acids (OA)*, where organic acids was calculated as *OA (mEq/d) = (body surface area (m*^*2*^*)*∗*41(mEq/d/1.73 m*^*2*^*))/1.73(m*^*2*^*)*[1]. The unit for all the three measures was mEq/day.

This calculation methodology, primarily based on PRAL, allows an appropriate prediction of the effects of diet on the acidity of urine. NAE provides an estimate of the production of the endogenous acid that exceeds the level of alkali produced for given amounts of food ingested daily. The method of calculation of NAE_es_ was experimentally validated in healthy adults, and it showed that acid loads and renal net acid excretion (NAE) can be reliably estimated from diet composition [1,2,21].

### Outcome

#### Measurement and classification of albuminuria and kidney function

Serum and urine samples were collected in the MEC. Serum creatinine was measured by means of the modified kinetic Jaffé method using different analyzers in different survey years. Random spot urine samples were obtained and frozen. Urine albumin was measured using solid-phase fluorescence immunoassay, and urine creatinine was measured using the modified Jaffé kinetic method in the same laboratory. Estimated GFR (eGFR) was calculated according to the isotope dilution mass spectrometry (IDMS)-traceable 4-variable MDRD Study equation for calibrated creatinine [17]. As specified in NHANES documentation [22], we corrected serum creatinine levels in the 1999–2000 survey. Albuminuria, which is calculated as the urinary albumin-to-creatinine ratio (ACR), is expressed as milligrams of albumin per gram of creatinine (mg/g Cr) using American Diabetes Association categories: normal (<30 mg/g Cr), and albuminuria (≥30 mg/g Cr) [23]. We defined the stages of CKD according to the National Kidney Foundation Kidney Disease Outcomes Quality Initiative (NKF KDOQI) CKD classification [24] based on the level of kidney function (eGFR) and presence or absence of kidney damage (albuminuria). According to the recent CKD nomenclature used by Kidney Disease Improving Global Outcome (KDIGO) [25], we defined the prognosis of CKD by eGFR and albuminuria categories where the risk groups ranged from low, moderately increased, high to very high.

#### Statistics

We considered participants for analysis who had complete data on their dietary recall interview. We compared participants with complete data on dietary intake and those with missing data on their dietary intake by *t*-test. Baseline characteristics of study participants across NAE_es_ quintiles were compared using *χ*^2^ tests for categorical and one-way ANOVA for continuous variables. Kruskal-Wallis test was used for the continuous variables if the normality assumption of the residuals was not met. As NAE_es_ had a skewed distribution, we performed median regression [26] to determine the association of participant characteristics with NAE_es_. Median regression specifies the changes in the median NAE_es_ as a function of the participant characteristics. Variables included in our models were demographics (age, gender, race/ethnicity), SES (education history, PIR), routine healthcare utilization, CKD risk factors (smoking, diabetes, hypertension, and cardiovascular disease), total caloric intake, and body mass index (BMI). The measure of diet-dependent acid load (i.e. NAE_es_) was divided into quintiles. We performed logistic regression analysis to assess the relation between quintiles of NAE_es_ and albuminuria, and eGFR <60 mL/min per 1.73 m^2^, both individually and with the inclusion of potential confounders by using the lowest quintile category as the referent. We calculated the *P* for trend across NAE_es_ quintiles by modeling them as continuous variables. Ordinal logistic regression was performed to analyze the relation between quintiles of NAE_es_ and the stages/risk groups of kidney disease, both individually and adjusted for the potential confounders as mentioned above. Because prior studies have demonstrated renal preservation effects of oral sodium bicarbonate in hypertensive patients, we conducted subgroup analyses of participants 1) with hypertension (and no diabetes) and 2) without hypertension (and no diabetes). Since both protein intake and phosphorus intake are likely to be independently associated with CKD, we performed sensitivity analyses to study the association of albuminuria and eGFR with protein intake and phosphorus intake independently using logistic regression. Sensitivity analyses with definition of reduced kidney function by the CKD-EPI equation were also performed [27]. Analyses included the dietary weights to account for the complex sample design of the survey. P < 0.05 was considered statistically significant. All analyses were performed using SAS 9.2 (SAS Institute, Inc. Cary, NC).

## Results

**Figure 2:**
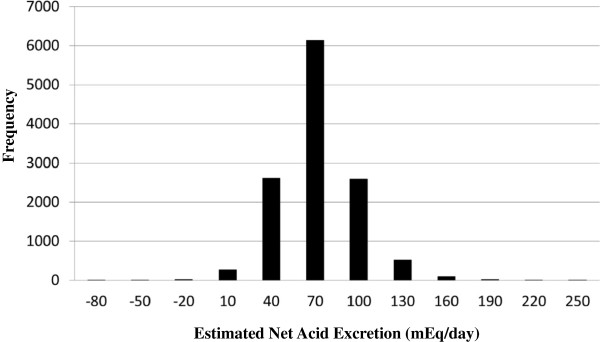
Frequency distribution of estimated Net Acid Excretion (from 24-hr dietary recall) in 12,293 NHANES (1999–2004) participants, median value (IQR) =55.15 (40.92, 71.07).

A total of 12,293 participants from NHANES 1999–2004 were included in this analysis. The mean age in this population was 49 years, 47.5% were males, 19.6% were NHB and 55.5% were NHW, 32% attended high school, 44.1% had PIR < 2, 21.6% were current smokers, 11.8% had diabetes, 33.5% had hypertension, 11% had any cardiac disease and the mean caloric intake/day was 2115 kcal/day. Out of the total, we had missing information for 20 participants regarding education history; PIR values were missing for 1008; smoking status was missing for 14; and history of cardiovascular disease was missing for 55 participants. There was no significant difference in the socio-demographic and clinical characteristics in the participants with complete dietary data (n = 12,293) whom we included in our study and those with missing dietary data whom we excluded (n = 875), except mean age (49 years in the included participants and 54 years in the excluded participants, P-value <0.001). The median value of NAE_es_, calculated using Remer and Manz formula (1), was 55.15 mEq/day (interquartile range 40.92, 71.07 mEq/day). The baseline participant characteristics across the quintiles of NAE_es_ are shown in Table 1. The distribution of NAE_es_ was skewed leftward (Figure 2).

**Table 1:** Characteristics of study participants by estimated Net Acid Excretion quintiles

**Characteristics**	**Net acid excretion (mEq/day)**					P- Value^*^
	**Quintile 1 (min- 37.35)**	**Quintile 2 (37.35–49.62)**	**Quintile 3 (49.62–60.89)**	**Quintile 4 (60.89–75.65)**	**Quintile 5 (75.65-max)**	
Age category (in years), (%)						<0.0001
20– < 40	717 (29.2)	834 (33.9)	845 (34.4)	887 (36.1)	1122 (45.6)	
40– < 60	776 (31.6)	654 (26.6)	719 (29.2)	758 (30.8)	774 (31.5)	
60– < 70	447 (18.2)	406 (16.5)	380 (15.4)	385 (15.7)	292 (11.9)	
≥70	517 (21.0)	565 (23.0)	516 (21.0)	429 (17.4)	270 (10.9)	
Age, continuous (mean ± SD)	47.1 ± 0.50	46.7 ± 0.61	46.9 ± 0.64	45.9 ± 0.48	42.9 ± 0.44	0.001
Gender, (%)						<0.0001
Male	879 (35.8)	1246 (50.7)	1407 (57.2)	1477 (60.1)	1442 (58.7)	
Female	1579 (64.2)	1213 (49.3)	1053 (42.8)	982 (39.9)	1015 (41.3)	
Race/Ethnicity, %						<0.0001
Non-Hispanic Blacks	381 (17.0)	342 (15.0)	395 (17.5)	458 (20.1)	639 (28.3)	
Mexican Americans	468 (20.9)	488 (21.4)	564 (25.0)	615 (27.1)	685 (30.3)	
Non-Hispanic Whites	1390 (62.1)	1450 (63.6)	1298 (57.5)	1201 (52.8)	937 (41.4)	
Poverty Income Ratio, %						<0.0001
≤2	848 (37.2)	880 (39.0)	923 (41.2)	1101 (48.4)	1228 (54.9)	
2– ≤ 3	389 (17.1)	335 (14.9)	386 (17.2)	338 (14.8)	365 (16.3)	
3– ≤ 4	278 (12.2)	326 (14.5)	315 (14.0)	268 (11.8)	229 (10.3)	
>4	762 (33.5)	713 (31.6)	618 (27.6)	570 (25.0)	413 (18.5)	
Education History, %						0.002
<High School	563 (22.9)	637 (25.9)	727 (29.6)	841 (34.3)	1104 (44.9)	
High School/Some College	1308 (53.3)	1269 (51.6)	1225 (50.0)	1201 (48.9)	1084 (44.2)	
>College	583 (23.8)	551 (22.5)	500 (20.4)	413 (16.8)	267 (10.9)	
Smoker, %						0.004
Current	570 (23.2)	527 (21.4)	456 (18.5)	511 (20.8)	594 (24.2)	
Past	647 (26.4)	695 (28.3)	689 (28.0)	664 (27.1)	607 (24.7)	
Never	1236 (50.4)	1235 (50.3)	1315 (53.5)	1280 (52.1)	1253 (51.1)	
Diabetes, %						0.11
Yes	284 (11.5)	298 (12.1)	305 (12.4)	302 (12.3)	254 (10.3)	
No	2175 (88.5)	2160 (87.9)	2155 (87.6)	2157 (87.7)	2203 (89.7)	
Hypertension, %						0.27
Yes	855 (34.8)	864 (35.2)	845 (34.3)	824 (33.5)	731 (29.8)	
No	1604 (65.2)	1594 (64.8)	1615 (65.7)	1635 (66.5)	1726 (70.2)	
Cardiac Disease, %						0.005
Yes	367 (15.0)	296 (12.1)	279 (11.4)	220 (9.0)	182 (7.4)	
No	2078 (85.0)	2146 (87.9)	2172 (88.6)	2231 (91.0)	2267 (92.6)	
Total Caloric Intake, %						<0.0001
<2000 Kcal/day	2172 (88.4)	1669 (67.9)	1068 (43.4)	613 (24.9)	322 (13.1)	
≥2000 Kcal/day	286 (11.6)	790 (32.1)	1392 (56.6)	1846 (75.1)	2135 (86.9)	

Poverty Income Ratio = a ratio of family income to poverty threshold.

HTN = Hypertension defined as self-reported, avg BP > 140/90 or use of medications.

Diabetes defined as self-reported or hemoglobin A_1c_ (A_1c_) ≥ 6.5%.

^*^By X^2^test for categorical variables and one-way ANOVA for continuous variables.

### Relation of NAE_es_ with albuminuria, eGFR, and CKD risk groups

#### Albuminuria

**Figure 3:**
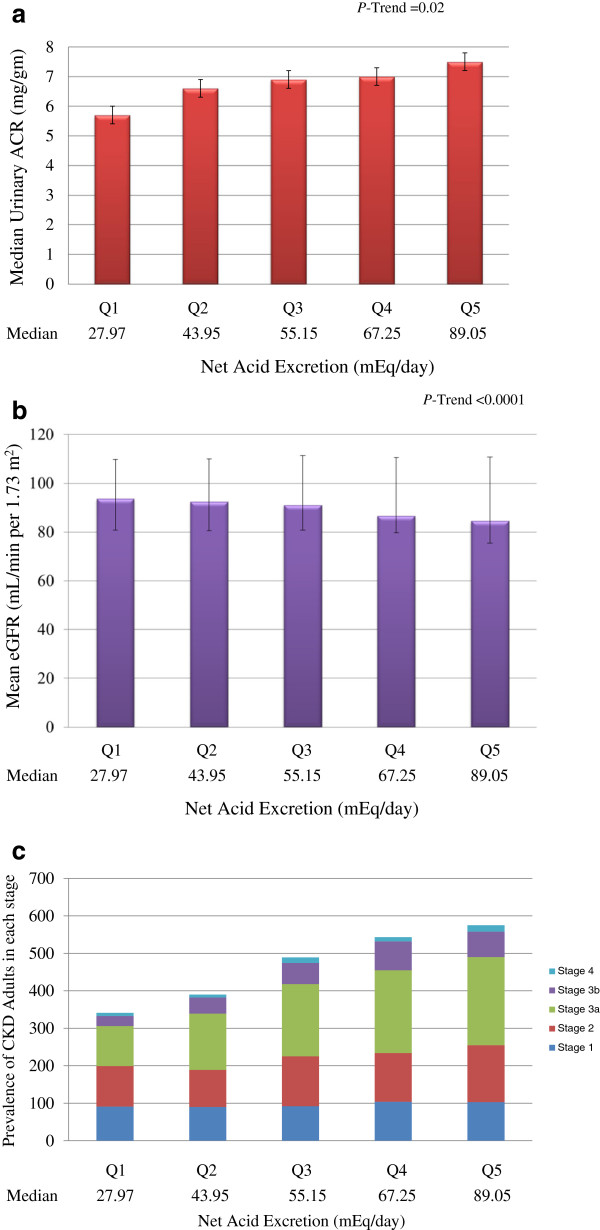
Distribution by the quintiles of estimated Net Acid Excretion of (a) Median Urinary Albumin-to-Creatinine Ratio (ACR) (b) Mean eGFR (c) Prevalence of CKD Adults in each Stage.

In our entire study population, participants with higher NAE_es_ had greater albuminuria (Figure 3a). While a significant trend of greater NAE_es_ with albuminuria was observed across quintiles after adjustment for potential confounders of demographics, SES, CKD risk factors, caloric intake, and BMI, the associations were significant just for the highest quintile (Table 2). Among participants with hypertension (Table 3), higher NAE_es_ was associated with the presence of albuminuria in unadjusted analyses. However, after multivariable adjustment for the potential confounders, there was no significant association of the quintiles of NAE_es_ with presence of albuminuria. Among participants without hypertension (Table 4), there was significantly greater NAE_es_ associated with albuminuria in unadjusted analyses. In multivariable analyses, only the highest quintile of NAE_es_ was significantly associated with albuminuria.

**Table 2:** Relationship of estimated Net Acid Excretion with Albuminuria, Kidney Dysfunction, and CKD Stages and Risk Groups in NHANES participants (N = 11,285)

**Parameter**	**UACR**^**+$**^ **> 30 mg/g**	**eGFR**^**$**^ **< 60 ml/min/1.73m**^**2**^	**Stages of CKD**^#,**^	**Risk groups of CKD**^@**^
	**OR (95% CI)**	**OR (95% CI)**	**OR (95% CI)**	**OR (95% CI)**
**Unadjusted**				
Quintile 1	Reference	Reference	Reference	Reference
Quintile 2	1.39 (1.09–1.78)	1.29 (0.98–1.70)	1.47 (1.11–1.95)	0.92 (0.76–1.11)
Quintile 3	1.33 (1.08–1.63)	1.84 (1.38–2.45)	1.79 (1.36–2.37)	1.30 (1.08–1.57)
Quintile 4	1.44 (1.15–1.80)	2.35 (1.78–3.12)	2.23 (1.63–3.05)	1.65 (1.41–1.93)
Quintile 5	1.71 (1.40–2.07)	2.63 (2.07–3.34)	2.51 (1.73–3.64)	1.99 (1.67–2.37)
**ptrend**	<0.0001	<0.0001	<0.0001	<0.0001
Adjusted*				
Quintile 1	Reference	Reference	Reference	Reference
Quintile 2	1.25 (0.80–1.94)	1.13 (0.89–1.44)	0.94 (0.52–1.70)	0.75 (0.60–0.94)
Quintile 3	1.36 (1.04–1.78)	1.06 (0.82–1.36)	1.10 (0.74–1.65)	0.92 (0.74–1.15)
Quintile 4	1.27 (0.98–1.66)	1.17 (0.86–1.58)	1.23 (0.91–1.65)	1.08 (0.88–1.29)
Quintile 5	1.57 (1.20–2.05)	1.37 (0.91–2.05)	1.20 (0.87–1.62)	1.10 (0.91–1.30)
**ptrend**	0.04	0.04	0.05	0.01

Quintile 1: min to 37.35 mEq/day; Quintile 2: >37.35 to 49.62 mEq/day; Quintile 3: >49.62 to 60.89 mEq/day; Quintile 4: >60.89 to 75.65 mEq/day; Quintile 5: >75.65 to max mEq/day.

^+^UACR- Urinary albumin to creatinine ratio.

^#^CKD Stages defined by KDOQI Classification.

^@^CKD Risk Groups defined by KDIGO Nomenclature.

*Adjustment for demographics (age, gender and race/ethnicity), socio-economic status (education, poverty income ratio), body mass index, risk factors (smoking, diabetes, hypertension, self-reported cardio-vascular diseases [CVD i.e. (coronary heart disease, congestive heart failure or stroke)]), total caloric intake, and body mass index.

OR (95% CI) = Odds Ratio (95% confidence interval).

^$^From multiple logistic regression.

^**^From ordinal logistic regression.

**Table 3:** Relationship of estimated Net Acid Excretion with Albuminuria, Kidney Dysfunction, and CKD Stages and Risk Groups in participants with hypertension but without diabetes (N = 3,204)

**Parameter**	**UACR**^**+$**^ **> 30 mg/g**	**eGFR**^**$**^ **< 60 ml/min/1.73m**^**2**^	**Stages of CKD**^#**^	**Risk groups of CKD**^@**^
	**OR (95% CI)**	**OR (95% CI)**	**OR (95% CI)**	**OR (95% CI)**
**Unadjusted**				
Quintile 1	Reference	Reference	Reference	Reference
Quintile 2	0.87 (0.53, 1.44)	1.38 (0.71, 2.31)	1.02 (0.75–1.40)	0.94 (0.70–1.28)
Quintile 3	0.83 (0.53, 1.28)	1.57 (0.99, 2.49)	1.37 (0.89–2.11)	1.32 (0.94–1.86)
Quintile 4	1.07 (0.65, 2.15)	2.21 (1.38, 3.55)	1.64 (1.12–2.39)	1.58 (1.12–2.23)
Quintile 5	1.30 (1.02, 2.27)	2.02 (1.31, 3.12)	2.51 (1.74–3.61)	2.22 (1.54–3.21)
**ptrend**	0.02	0.02	0.001	<0.0001
Adjusted*				
Quintile 1	Reference	Reference	Reference	Reference
Quintile 2	0.94 (0.49–1.80)	1.43 (0.96–2.14)	0.65 (0.58–1.14)	0.75 (0.55–1.02)
Quintile 3	1.47 (0.75–2.85)	1.59 (0.96–2.63)	1.00 (0.60–1.58)	0.98 (0.65–1.41)
Quintile 4	1.11 (0.69–1.76)	1.75 (0.92–3.31)	0.79 (0.43–1.30)	0.81 (0.55–1.20)
Quintile 5	1.31 (0.90–1.91)	1.75 (1.11–2.75)	1.11 (0.56–2.91)	1.00 (0.61–1.55)
**ptrend**	0.04	0.01	0.10	0.08

Quintile 1: min to 37.35 mEq/day; Quintile 2: >37.35 to 49.62 mEq/day; Quintile 3: >49.62 to 60.89 mEq/day; Quintile 4: >60.89 to 75.65 mEq/day; Quintile 5: >75.65 to max mEq/day.

^+^UACR- Urinary albumin to creatinine ratio.

^#^ CKD Stages defined by KDOQI Classification.

^@^CKD Risk Groups defined by KDIGO Nomenclature.

*Adjustment for demographics (age, gender and race/ethnicity), socio-economic status (education, poverty income ratio), body mass index, and risk factors (smoking, diabetes, hypertension, self-reported cardio-vascular diseases [CVD i.e. (coronary heart disease, congestive heart failure or stroke)]), total caloric intake, and body mass index.

OR (95% CI) = Odds Ratio (95% confidence interval).

^$^From multiple logistic regression.

^**^From ordinal logistic regression.

**Table 4:** Relationship of estimated Net Acid Excretion with Albuminuria, Kidney Dysfunction, and CKD Stages and Risk Groups in participants without hypertension and without diabetes (n = 6,772)

**Parameter**	**UACR**^**+$**^ **> 30 mg/g**	**eGFR**^**$**^ **< 60 ml/min/1.73m**^**2**^	**Stages of CKD**^#,**^	**Risk groups of CKD**^@**^
	**OR (95% CI)**	**OR (95% CI)**	**OR (95% CI)**	**OR (95% CI)**
**Unadjusted**				
Quintile 1	Reference	Reference	Reference	Reference
Quintile 2	1.12 (0.77, 1.64)	1.26 (0.93, 1.69)	1.57 (1.11–2.22)	0.96 (0.75–1.23)
Quintile 3	1.62 (1.09, 2.39)	1.79 (1.23, 2.59)	1.88 (1.22–2.90)	1.64 (1.34–2.01)
Quintile 4	1.57 (1.16, 2.14)	2.09 (1.46, 2.99)	3.29 (2.06–5.27)	1.93 (1.51–2.46)
Quintile 5	1.79 (1.21, 2.65)	2.52 (1.80, 3.52)	3.51 (1.94–6.36)	2.99 (2.29–3.88)
**ptrend**	0.04	<0.0001	<0.0001	<0.0001
Adjusted*				
Quintile 1	Reference	Reference	Reference	Reference
Quintile 2	0.96 (0.58–1.58)	0.82 (0.44–1.51)	1.26 (0.66–2.40)	0.77 (0.55–1.06)
Quintile 3	1.23 (0.80–1.88)	0.85 (0.58–1.25)	1.27 (0.70–2.31)	1.03 (0.77–1.37)
Quintile 4	1.09 (0.72–1.63)	1.07 (0.78–1.45)	1.13 (0.72–1.78)	0.90 (0.63–1.28)
Quintile 5	1.58 (1.04–2.39)	1.42 (1.07–2.10)	1.41 (0.92–2.16)	1.12 (0.75–1.67)
**ptrend**	0.05	0.01	0.06	0.07

Quintile 1: min to 37.35 mEq/day; Quintile 2: >37.35 to 49.62 mEq/day; Quintile 3: >49.62 to 60.89 mEq/day; Quintile 4: >60.89 to 75.65 mEq/day; Quintile 5: >75.65 to max mEq/day.

^+^UACR- urinary albumin to creatinine ratio.

^#^CKD Stages defined by KDOQI Classification.

^@^CKD Risk Groups defined by KDIGO Nomenclature.

*Adjustment for demographics (age, gender and race/ethnicity), socio-economic status (education, poverty income ratio), body mass index, and risk factors (smoking, diabetes, hypertension, self-reported cardio-vascular diseases [CVD i.e. (coronary heart disease, congestive heart failure or stroke)]), total caloric intake, and body mass index.

OR (95% CI) = Odds Ratio (95% confidence interval).

^$^From multiple logistic regression.

^**^From ordinal logistic regression.

#### Reduced eGFR

We found participants with greater NAE_es_ had lower eGFR in our analysis (Figure 3b). In unadjusted analyses, the fifth quintile of NAE_es_ was associated with 2.6 fold greater odds of kidney dysfunction as compared with the first quintile. After multivariable adjustment for demographics, SES, CKD risk factors, caloric intake, and BMI, a significant trend of greater NAE_es_ being associated with reduced eGFR persisted (Table 2). The highest quintile of NAE_es_ was associated with 1.4 fold greater odds of low eGFR compared with the lowest quintile. Among participants with hypertension (but without diabetes), in unadjusted analyses, the highest quintile of NAE_es_ was associated with 2 fold greater odds of kidney dysfunction (low eGFR) as compared with the lowest quintile. The strength of the association of NAE_es_ with kidney dysfunction though was attenuated after adjustment for the confounders but the association remained significant (Table 3). Among participants without hypertension (and without diabetes), there was a statistically significant trend of an association of greater NAE_es_ with reduced eGFR. The highest quintile of NAE_es_ was associated with 1.4 fold greater odds of kidney dysfunction compared with the lowest quintile (Table 4).

#### Risk groups of CKD

Figure 3c shows the proportion of adults in the advanced stages of CKD (as defined by National Kidney Foundation Kidney Disease Outcomes Quality Initiative (NKF KDOQI) CKD classification [20]) with the increasing NAE_es_. When we examined the Kidney Disease Improving Global Outcome (KDIGO) [21] CKD risk groups, we found in multivariable analyses, highest quintile of NAE_es_ had 1.1 times greater odds of more severe risk of CKD than the lowest quintile (Table 2). We found a significant trend of greater NAE_es_ with severity of CKD (p = 0.01). Unadjusted analysis in the subgroup of participants with hypertension showed participants in the highest quintile of NAE_es_ had 2 times greater odds of more advanced CKD stages compared with the lowest quintile, with a significant trend across quintiles (P-trend <0.0001) (Table 3). When the model was adjusted for age, there was no statistically significant association between the highest quintile of NAE_es_ and increasing risk of advanced CKD stages compared with the lowest quintile (OR [95% CI]: 1.27 [0.84–1.91]). When adjusting the model for the other confounders, the odds ratios further attenuated (Table 3). Among participants without hypertension, in the multivariable analysis, the odds of having more advanced CKD stages with the highest quintile was 1.1 times compared with the lowest quintile (Table 4).

### Relation of participant characteristics to NAE_es_

**Figure 4:**
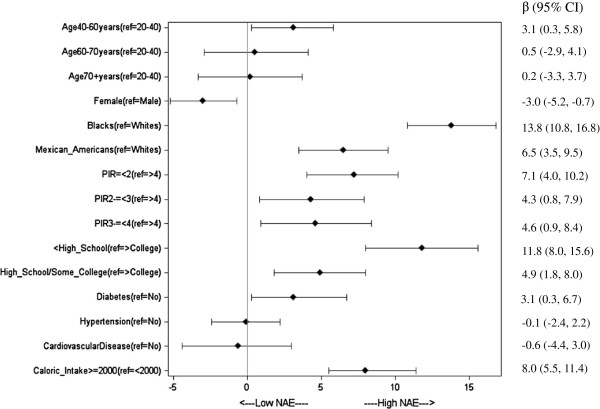
**Association of NHANES (1999–2004) Participant Characteristics with estimated Net Acid Excretion (NAE).** The graph shows adjusted beta coefficients, from quintile (median) regression. Points show beta coefficients per quintile, and bars show 95% confidence intervals. Reference groups (Dots on the vertical line = 0) are not shown for clarity.

In unadjusted analyses there were greater NAE_es_ values among younger adults, males, and racial/ethnic minorities. Multivariable analysis showed significantly greater NAE_es_ among 40–60 yearr olds and male participants (Figure 4). NHBs and Mexican Americans had significantly higher NAE_es_ than NHWs. Higher NAE_es_ was observed in the least educated and lowest income participants (less than 200% of federal poverty line) compared to their more educated and higher income counterparts. Unadjusted analysis showed current smokers and participants with a previous history of smoking had lower NAE_es_ than those who had never smoked but there was no significant difference across these groups. We did not find a significant association of NAE_es_ with healthcare utilization in unadjusted analysis. In unadjusted analyses, higher NAE_es_ was present among participants with diabetes, hypertension, or cardiovascular disease as compared to those without these conditions; however in multivariable analyses, only cardiovascular disease reached statistical significance.

### Sensitivity analysis

#### Relation of protein and phosphorus intake with albuminuria and eGFR

To study the influence of protein and phosphorus intake on kidney disease, we analyzed the association between albuminuria and protein intake and phosphorus intake independently. Our results did not find a statistically significant association between protein intake and albuminuria in multivariable analysis, but a trend toward significance was observed for the higher values of protein intake with albuminuria (P-trend = 0.02). We did not observe a significant association with the phosphorus intake and presence of albuminuria as well. On analyzing the association of eGFR with protein and phosphorus intake, the odds of lower eGFR continued to be statistically non-significant in the multivariable model.

#### Kidney function defined by CKD-EPI equation

The odds associated with lower eGFR and the risk groups of CKD defined by CKD-EPI equation in NHANES participants did not differ substantially from the odds estimated using the MDRD Study equation (data not shown). When the model was adjusted for the potential confounders, there was again no substantial change in the results (data not shown). Subgroup analyses of participants also showed that the odds of having more advanced CKD stages with the highest quintile compared with the lowest quintile were not different from the odds obtained by using the MDRD equation (OR [95% CI]: 1.10 [0.64–1.87] in participants with hypertension and 1.30 [1.02–1.75] in participants without hypertension).

## Discussion

In this nationally representative cohort, we found that greater DAL, quantified by estimated NAE was associated with albuminuria among U.S. adults. Moreover, several socio-demographic factors (age 40–60 years, low income, and racial/ethnic minority status) were independently associated with high DAL.

Western diets, which are generally high in animal and grain products, are high in acid precursors. In general, common foods that impart a high DAL include cheese, meat, eggs, and grains; whereas fruits and vegetables provide alkali [1,28]. The average American consumes approximately 15–17% of their energy as protein, predominantly from animal sources [29]. In addition, it is low in potassium-rich fruit and vegetables [30], resulting in an average DAL of approximately 1 mEq/kg/day [31]. Our findings raise the possibility of high acid containing diets playing a role in the development of CKD. They buttress the findings of smaller studies that have shown that dietary acid reduction with either NaHCO_3_ and/or base-inducing fruits and vegetables slow GFR decline and reduce urinary parameters of kidney injury in CKD patients [32]. While this area is still being developed, there are studies that have suggested that high dietary acid load might mediate progressive GFR decline in those with moderately reduced GFR due to hypertensive nephropathy [7,11]. Our results not only suggest higher risk of albuminuria and low eGFR in participants with hypertension but a similar magnitude effect was observed for participants without hypertension or diabetes.

In our study, we examined the association between protein intake and phosphorus intake with albuminuria. Although there is concern that high protein intake and phosphorus intake may promote renal damage by chronically increasing glomerular pressure and hyperfiltration [33-35], we did not find a significant association between them. This is not surprising in that Remer et al. [1,2], Goraya et al. [8,32], and Scialla et al. [36] show that the more important determinant of the effect of dietary protein on nephropathy progression is the quality of the ingested protein (i.e., whether it induces acid-production [like most animal protein] or base production [like most fruit and vegetable protein]) when ingested rather than the quantity of protein ingested. It is important to note that our results suggested a higher risk of albuminuria in adults with greater DAL than that suggested by the amount of either protein or phosphorus intake alone. A balance of protein intake with base-inducing fruits and vegetables may be important for containing renal damage.

Our finding of an association between low socioeconomic-status (SES) and greater NAE_es_ is intriguing. Lower financial means and living in certain communities may affect the ability of individuals to obtain a diet rich in fruits and vegetables. Studies of the food and low SES environment suggest that low-income individuals often live in neighborhoods where there are few full-service grocery stores and may not have easy access to transportation to allow for shopping at such stores in outlying areas [37]. Limited access to nutritious food and relatively easier access to less nutritious food may be linked to poor diets and, ultimately, to diet-related diseases. Differences in access to food may create structural barriers in poor communities that lead to poor health behaviors and health inequalities [38].

Fresh fruits and vegetables can be expensive, and low-income individuals must weigh how they will use their scarce resources. Prior studies found that having a lower income and no greater than a high school education were associated with consumption of fewer servings of fruits and vegetables per day [39,40]. Energy-dense foods composed of refined grains, added sugars, or fats may represent the lowest-cost option [41,42]. The consumption of more fruits and vegetables typically lowers the DAL [32,43]. In contrast, sugar consumption has been linked with an increase in uric acid levels, which in turn may promote kidney damage [44]. Thus, low SES might lead to greater CKD burden via this pathway [44,45].

Although our findings are consistent with some prior studies [5-8] suggesting that the risk for CKD or its progression may be mitigated by a reduction in the DAL, this area remains controversial. Jara et al. found that the combination of chronic metabolic acidosis and phosphate loading in azotemic rats may protect against the progression of renal failure, because the harmful effects of acidosis and phosphate loading may be counterbalanced [46]. In a similar study, Mendoza et al. [47] found that metabolic acidosis inhibits vascular and soft-tissue calcifications in calcitriol-treated uremic rats. Since acidosis prevents upregulation of vascular Pit-1 expression, a possible mechanism for its anticalcifying effect may be reduced cellular uptake of phosphate [47].

Despite the strengths of a large study representative of the U.S. population, the detailed collection of nutritional parameters, and standardized laboratory testing that is integral to the design of the NHANES, our study had certain limitations. First, it was an observational study, and although we adjusted for potential confounders associated with both diet content and CKD, residual confounding is likely. Second, by design, the NHANES is cross-sectional; therefore, causality cannot be inferred and there is a possibility of misclassification of risk factors for CKD such as diabetes and hypertension which are defined from measurements at a single time point. Third, we lacked a measure of the neighborhood food environment which may affect individual dietary patterns [48]. We also did not assess health behaviors that may be closely associated with dietary practices, such as physical activity and adherence to medications. Fourth, we did not have any details available on the family history of kidney disease for these participants. Fifth, a 24-hour dietary recall was used to assess the usual intake which is subject to bias. However, previous literature has demonstrated the reliability of dietary intake measures from the 24-hour dietary recalls [49]. Sixth, we analyzed participants for whom we had complete data of their dietary recall interview, thus introducing the potential for selection bias. However, there was no significant difference in the socio-demographic and clinical characteristics in the participants whom we included in our study and those excluded, except age. Finally, we estimated NAE_es_ from dietary data using previously validated equations rather than directly measuring NAE_es_ in participants’ urine.

## Conclusion

To our knowledge, this is one of the few studies to assess the relationship of dietary renal acid load with markers of CKD in a large, representative population, and to examine the association of socio-demographic characteristics with dietary renal acid load. Our findings suggest that high DAL is associated with greater risk of markers of CKD, and older age, poverty, racial/ethnic minority status, and limited education are independently associated with high DAL among U.S. adults. The findings have important implications, in that, if they are corroborated in other studies, altering diets may provide an adjunct approach to other strategies for treatment of CKD. Longitudinal studies in large representative populations should be conducted to examine a potential causal relationship between NAE_es_ and CKD.

## Competing interests

All the authors disclosed no competing interests.

## Authors' contributions

TB was responsible for concept, analytic design, data analysis, interpretation of results, and manuscript preparation. DC and NP participated in the concept, analytic design, data analysis, interpretation of results, and preparation of manuscript. DW gave thoughtful comments on an early version of this manuscript, helped with the interpretation of results, and critically reviewed the manuscript. AT, RS, NRB, and DW helped with the interpretation of results, gave thoughtful comments, and critically reviewed the manuscript. All authors read and approved the final manuscript.

## Pre-publication history

The pre-publication history for this paper can be accessed here:

http://www.biomedcentral.com/1471-2369/15/137/prepub
